# Selenium Deficiency in COVID-19—A Possible Long-Lasting Toxic Relationship

**DOI:** 10.3390/nu14020283

**Published:** 2022-01-11

**Authors:** Lutz Schomburg

**Affiliations:** Institute for Experimental Endocrinology, Charité-Universitätsmedizin Berlin, Corporate Member of Freie Universität Berlin, Humboldt-Universität zu Berlin, and Berlin Institute of Health, 10115 Berlin, Germany; lutz.schomburg@charite.de; Tel.: +49-30-450-524-289; Fax: +49-30-450-752-4289

In the last two years, there has been a surge in the number of publications on the trace element selenium (Se) and selenocysteine-containing selenoproteins in human health, largely due to the pandemic and the multiple roles that this micronutrient and Se-dependent selenoproteins play in various aspects of the disease. In early 2020, two publications increased the attention on Se status in the context of SARS-CoV-2, namely, a population-based analysis correlating cure rates with baseline Se status in different regions of China [1], and an analysis of individual patients who did or did not survive COVID-19 [2]. Both studies indicated that Se deficiency was a risk factor for severe disease progression, poor recovery, and eventually COVID-19-related death. Soon, these findings were supported by several observational studies, e.g., on regional Se supply and severity of COVID-19 disease [3], on patients with comorbidities [4], or on patients with a parallel deficiency in zinc, the second most important immune-relevant trace element [5,6,7]. Overall, a picture emerged in which Se is an important parameter for COVID-19 disease progression and survival, and severe Se deficiency measured in blood serving as a potential indicator of target cell Se deficiency associated with organ system dysfunction and death (Figure 1A).

These results were not unexpected, as previous studies had pointed out the importance of Se status for the immune system and survival in critical illness [8]. The most solid data base for this essential link was provided by several studies in intensive care units, where Se deficiency was found to be an important predictor of severe disease course and sepsis-related mortality [9,10,11]. The underlying causes are not yet clear, but the negative acute-phase response of liver-derived selenoprotein P (SELENOP) and serum Se concentrations as biomarkers of poor systemic Se transport and target organ delivery appear to be related to the observed association between low circulating Se concentrations and unfavorable prognosis [12,13]. The strong decline in serum Se and SELENOP may directly lead to worsening disease, particularly in relation to the immune system [14], the endocrine system [15] and the central nervous system [16,17], where insufficiently low Se concentrations cause functional defects that can manifest in specific disease symptoms (Figure 1B).

It has been observed that severe Se deficiency due to pregnancy, inflammation or other conditions directly increases autoimmune disease risk [18,19,20]. The most conclusive data for this notion were obtained by comparing the incidence of autoimmune thyroid disease as a function of Se status [21], or thyroid disease prevalence in areas with low versus moderate baseline Se intake [22]. These observational findings have recently been supported by a focused molecular analysis of the role of the selenoprotein GPX4 in a specific severe autoimmune disease, i.e., systemic lupus erythematodes, in which downregulation of GPX4 gene transcription contributes to neutrophil ferroptosis as a key driver of neutropenia [23]. Similarly, humoral immunity has been shown to depend on follicular helper T-cells, which may undergo ferroptosis when GPX4 expression is low, leading to impaired immune responses to infection or vaccination in Se deficiency [24]. Importantly, these effects have been shown to be directly Se-dependent and seem preventable by Se supplementation, as demonstrated, e.g., in a well-conducted randomized controlled trial in pregnant women at risk of postpartum thyroiditis or by supplementation in COVID-19 patients, suggesting overall that severe Se deficiency is an addressable risk factor for long-term complications [25,26]. Whether this relationship also applies to COVID-19 and the long-COVID symptoms is not known, but currently seems plausible and requires experimental proof [20]. The observed Se deficiency in patients with COVID-19 may be secondary to increased inflammatory tone [27,28,29], hypoxia [30], and organ dysfunction [31,32,33], which develop in parallel and appear to worsen in those patients with poor prognosis [2].

Adequate Se status is not only a prerequisite for proper immune system functioning [34,35], but also of great importance for the central nervous system [16,36,37] and the endocrine control of energy and carbohydrate metabolism [38,39,40]. Pioneering work in some refined rodent model systems has shown that excess Se intake or transgenic overexpression of selenoproteins can impair glucose control, induce diabetes-like symptoms, and increase the risk of hyperglycemia [41,42]. Disruption of the regular peroxide-dependent signaling cascades by increased peroxidation appears to contribute to these defects [43,44]. Accordingly, a relationship between serum Se and insulin resistance has been reported from several observational studies. Thus, high Se status could impair normal insulin sensitivity, or conversely, circulating Se levels could be elevated in diabetes due to insulin resistance and elevation of hepatic SELENOP biosynthesis as a meaningful measure to protect from elevated circulating glucose levels. The latter explanation seems to be valid [45,46], as no increased type 2 diabetes mellitus risk was found in individuals taking Se supplements in the large supplementation studies [47]. Recent data from the NHANES III study, which analyzed the Se status of diabetic patients, suggest that serum Se concentrations correlate positively with life expectancy and protection from cardiovascular events [48]. Nevertheless, caution is mandatory as excessive supplemental Se intake can be toxic [49], and the use of very high therapeutic Se dosages may exceed the endogenous safety regulatory pathways that normally limit SELENOP biosynthesis to a certain maximum [50]. This may result in an oversupply of Se to target cells, which impairs regular peroxide-dependent signaling and leads to impaired control of insulin biosynthesis and insulin resistance.

The Se-dependent disruption of homeostatic control of carbohydrate metabolism can also be observed in the other direction, i.e., in Se deficiency. Marginal Se intake was linearly associated with hypoglycemia in a large observational study in China [51]. This surprising result provided a possible molecular and metabolic cause for poor survival in severe disease-related Se deficiency. Moreover, this notion accords with prior research on the association between Se deficiency as a risk factor for heart failure, cardiac events, and death from cardiovascular disease [52,53,54,55,56]. Therefore, new tools are needed to identify patients at risk for Se deficiency at an early stage, especially since Se intake cannot be reliably predicted or inferred from dietary habits [57,58]. Overall, the biological derangements resulting from severe Se deficiency are diverse and complex because multiple organ systems are involved, which interact, and the disorders develop dynamically (Figure 2).

Informative transgenic mouse models have highlighted the specific roles of individual selenoproteins causing some severe developmental, neurological, immunological, or metabolic phenotypes [59,60,61]. Similarly, rare human inherited diseases affecting regular selenoprotein biosynthesis have pinpointed some most relevant factors implicated in selenoprotein biosynthesis, and highlighted their association with developmental disorders, neuronal defects, infertility or the disruption of other regulatory pathways [62,63,64]. However, the problems we currently observe with COVID-19 rather result from acquired deficiencies due to the inflammatory disease and do not represent inherited or chronic defects. A similar severe decrease in circulating Se concentration has been observed before in severe sepsis [9] or at the end of pregnancy in areas of low Se supply [65]. Of note, all three conditions, i.e., COVID-19, sepsis and pregnancy, are associated with long-term sequelae and slow full recovery. It can take years to overcome sepsis [66], and similarly, pregnancy can directly cause permanent autoimmune disease [67,68] as well as chronic postpartum depression [69,70]. The supportive effects of supplemental administration of Se on mortality, progression, and recovery from sepsis are currently unclear [71], but positive studies have been reported [10]. Experience with Se in pregnancy is more consistent, and supplementation has been effective in preventing postpartum thyroiditis and postpartum depression [25,72,73]. The extent to which this experience can be extrapolated to COVID-19 and long-term impairment remains to be tested, but at least from experience, potential success in preventing severe Se deficiency during illness and convalescence seems likely, while adverse effects have not been reported when Se-deficient individuals were given adequate amounts of Se [46].

Knowledge of the role of Se in cancer, cardiovascular disease, and the other major non-communicable diseases is relatively solid and will expand further, in particular with respect to ageing [74,75,76], and by analytical studies applying several biomarkers of Se status in parallel [40,77,78]. Other important areas of future research include a better understanding of the Se status for fertility and early development, and the mechanisms of Se transport [31]. Recent reports indicate unexpectedly high variability of trace elements in the follicular fluid surrounding the maturing oocytes prior to ovulation [79], a direct relationship between Se concentration in amniotic fluid and development of small-for-gestational-age newborns [80], and an increased risk of a number of pregnancy complications in women with low Se levels in the first trimester [81]. The importance of iodine and thyroid hormones in these conditions and their homeostatic control is well established, and it seems imperative to give equal attention to the Se status.

Another urgent, remarkable, and well-characterized interaction between Se deficiency and COVID-19 involves the virus itself, i.e., its variability and mutation frequency. In some very elegant experiments, Melinda A. Beck’s group has demonstrated that Se-deficient host organisms provide a perfect environment for enhanced viral mutagenesis. Mice with low Se and vitamin E status or deficient of intracellular cytosolic glutathione peroxidase (GPX1) exhibited increased mutation rates of infectious viruses, allowing apathogenic viruses to evolve into highly pathogenic viral quasispecies, likely due to increased reactive oxygen species tone and consequent damages [82,83]. The extent to which Se deficiency in chronically ill, immunosuppressed or poorly supplied individuals contributes to the evolution of new variants of concern of the current SARS-CoV-2 virus remains to be investigated in the coming months [84]. Thus, again, efforts should be made to avoid severe Se deficiency on a population-wide scale and in individual at-risk patients to reduce the likelihood of increased mutagenesis and spread of new virus variants. However, it is unlikely that this can be achieved quickly and that the contribution of severe Se deficiency to pandemic spread and emergence of new viral strains can be directly attributed to low Se and causality demonstrated. From a personal point of view, actively avoiding Se deficiency through a wisely chosen diet or the use of dietary supplements seems to be a most sensible and efficient measure to reduce one’s health risks, to improve survival chances in disease and to avoid long-term sequelae after infection.

## Figures and Tables

**Figure 1:**
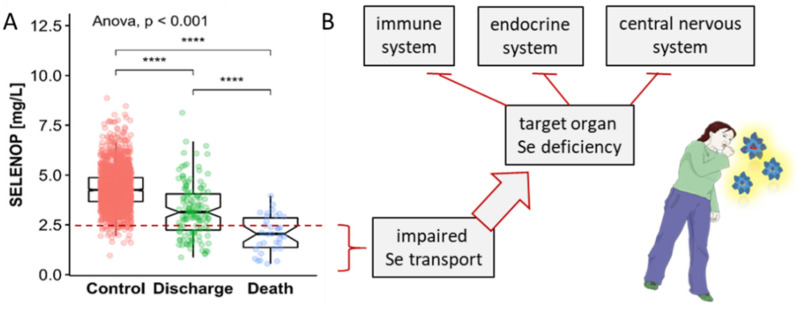
Relationship between serum concentrations of the Se transporter selenoprotein P (SELENOP) and survival in COVID-19 and possible implications for Se supply to target organs and systemic defects. (**A**) Patients with COVID-19 display strongly reduced SELENOP concentrations compared to healthy adults (Controls). In particular, non-survivors (Death) showed very severe Se deficiency compared to survivors who left the hospital alive (Discharge). The red broken line indicates the 2.5th percentile of serum SELENOP as indicator of severe Se deficiency. (**B**) Severely reduced serum SELENOP concentrations indicate decreased Se transport to target organs, resulting in partial Se deficiency in the three major communication systems of the human organism, i.e., the immune, the endocrine and the central nervous system. The survival data have been published [2]. Spearman’s correlation test was applied (2-sided, 2-tailed), **** indicates *p* < 0.0001.

**Figure 2:**
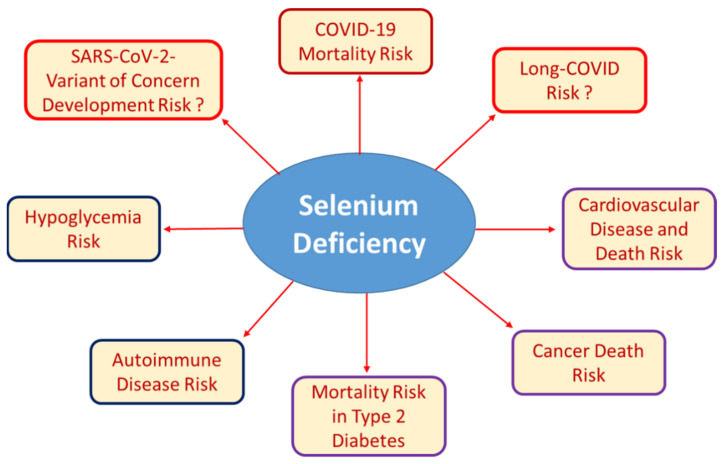
Well-established and recently described associations of Se deficiency with certain health risks. Low Se status can result from insufficient nutritional supply, chronic disease, inflammation, infection, or as a result from pregnancy, surgery or other conditions of increased need. Severe Se deficiency is known to be associated with autoimmune disease and increased risk of mortality due to cancer, cardiovascular disease, or infection. Recent research has added hypoglycemia risk and poor survival of patients with COVID-19 or type 2 diabetes mellitus to the list of Se-dependent health issues. The importance of poor Se status for the development of SARS-CoV-2 variants of concern due to increased mutagenesis in Se-deficient hosts or for the development and resolution of long-COVID symptoms are other potential links, but respective studies and relevant data are not yet at hand.
